# [^18^F] Sodium Fluoride PET Kinetic Parameters in Bone Imaging

**DOI:** 10.3390/tomography7040071

**Published:** 2021-12-01

**Authors:** Tanuj Puri, Michelle L. Frost, Gary J. Cook, Glen M. Blake

**Affiliations:** 1 Department of Biomedical Engineering, School of Biomedical Engineering and Imaging Sciences, King’s College London, London SE1 7EH, UK; tanujpuri82@gmail.com; 2 Institute of Cancer Research Clinical Trials & Statistics Unit (ICR-CTSU), Institute of Cancer Research, Sutton SM2 5NG, UK; michelle.frost@icr.ac.uk; 3 Department of Cancer Imaging, School of Biomedical Engineering and Imaging Sciences, King’s College London, London SE1 7EH, UK; gary.cook@kcl.ac.uk

**Keywords:** dynamic positron emission tomography, PET, computed tomography, CT, [^18^F] sodium fluoride, [^18^F]NaF, Hawkins model, kinetic modeling, bone metabolism, clinical significance

## Abstract

This report describes the significance of the kinetic parameters (k-values) obtained from the analysis of dynamic positron emission tomography (PET) scans using the Hawkins model describing the pharmacokinetics of sodium fluoride ([^18^F]NaF) to understand bone physiology. Dynamic [^18^F]NaF PET scans may be useful as an imaging biomarker in early phase clinical trials of novel drugs in development by permitting early detection of treatment-response signals that may help avoid late-stage attrition.

## 1. Introduction

The assessment of skeletal metabolism is important for understanding the pathophysiology and for measuring the response to treatment of metabolic bone diseases such as osteoporosis [1] and for investigating and differentiating the different effects of chronic kidney disease on bone [2]. Bone biopsy is considered the gold standard for measuring bone turnover; nonetheless, it is limited to a single biopsy site at the iliac crest, subject to large measurement errors and is invasive and uncomfortable for the patient [2,3,4,5,6,7,8] Moreover, multiple biopsies are required to assess treatment response and disease progression. The most commonly used and practical method is the measurement of bone turnover markers in serum and urine, which can show a large and rapid response within weeks of the commencement of treatment for osteoporosis [9,10]. However, they only provide information about global skeletal bone turnover throughout the entire skeleton [11,12,13,14]. Hence, the use of the noninvasive functional imaging technique of positron emission tomography (PET) using [^18^F] sodium fluoride ([^18^F]NaF) tracer to understand regional bone physiology, such as bone formation and perfusion [15], is attractive. The use of [^18^F]NaF has been validated by comparison with measurements from bone biopsy [2,16], and therefore has the potential to be used as an imaging biomarker for testing new hypotheses in clinical trials and research studies or as a clinical decision-making tool for use in healthcare [17] after standardization via multicenter trials.

The fluorine18-fluoride ion is an excellent bone imaging tracer that is extracted by the skeletal system in proportion to bone blood flow and bone metabolism at the sites of newly formed bone. A tracer is a substance that traces a physiologic or biochemical process. Tracers are small or larger molecules (e.g., antibodies or peptides) that are labeled with radionuclides. The labeled molecules are called radiotracers or radiopharmaceuticals [18]. The use of the fluorine18-fluoride ion as a bone imaging tracer was first introduced by Blau et al. [19]. Other bone-seeking radiotracers include the technetium-99m diphosphonate bone scan agents and radionuclides of calcium and strontium, such as ^47^Ca and ^85^Sr formerly used for tracer kinetic studies [20]. There are no radionuclides of calcium or strontium with gamma ray or positron emissions suitable for imaging studies, but even if there were, the slow clearance of these elements from soft tissue due to protein binding and high renal tubular reabsorption (Ca: 99%; Sr: 97%) renders them unsuitable for this application. The gamma camera bone scan agent technetium-99m methylene diphosphonate ([^99m^Tc]MDP) also shows significant protein binding (20% soon after injection, rising to 60% by 4 h), but free (i.e., unbound) [^99m^Tc]MDP is cleared through the kidneys by glomerular filtration and this aids the rapid clearance of tracer from soft tissue facilitating a clear image of the skeleton by 4 h after injection [21,22] As a bone imaging agent, [^18^F]NaF has the advantage of a higher clearance rate to bone than [^99m^Tc]MDP and no protein binding to delay renal clearance from soft tissue [20], resulting in high quality bone scan images at 1 h after injection. There is some degree of reabsorption in the kidneys that varies with urine flow rate, but this is not a serious problem in a well-hydrated patient [20,21].

Bone-seeking tracers are taken up by the newly forming hydroxyapatite crystals at sites of bone formation [23,24,25]. The quantitative uptake of these tracers measured on images is also sometimes referred to as bone turnover or bone remodeling, even though the two processes are not the same [26]. Bone remodeling is an active process referring to the metabolic activity in the bone, characterized by a tightly coupled cyclic process between osteoblasts, osteoclasts, and osteocytes to remove old bone and replace it with new [27,28,29]. Bone remodeling increases in women after menopause, with an increase in osteoclast activity and a decrease in bone formation rate (BFR) and osteoblast activity. On the other hand, bone turnover is defined based on bone formation rate and/or activation frequency [30,31], and considering the net effect of remodeling on bone volume that is both resorbed and formed over a period of time [32]. Low turnover is associated with reduced osteoblast and osteoclast activities, and high bone turnover is associated with increased osteoblast and osteoclast activities, for example, in adynamic and hyperparathyroid bone diseases, respectively [3]. In adults, bone turnover occurs mainly through bone remodeling [30].

The noninvasive imaging technique of [^18^F]NaF PET can measure early response to treatment (within weeks of treatment commencement) at clinically important sites for novel drugs being developed for osteoporosis [33,34,35,36,37] or other metabolic bone diseases [2,3,38] and therefore can help to avoid late-stage attrition during the drug development process. It has also been used to compare bone perfusion or turnover at different clinically important skeletal sites with varying amounts of trabecular and cortical bone, to examine the fracture healing processes [39] and investigate response to treatment in metastatic bone disease [40]. The methods of measuring bone metabolism have been extended in recent years. K_i_ values can be obtained from whole-body static PET images acquired for diagnostic purposes in the clinic [41], as well as from dynamic scans as short as 12 min long [42] that have the potential for translation to the clinic. A recent review by Blake et al. [43] provides more details in this context.

**Figure 1:**
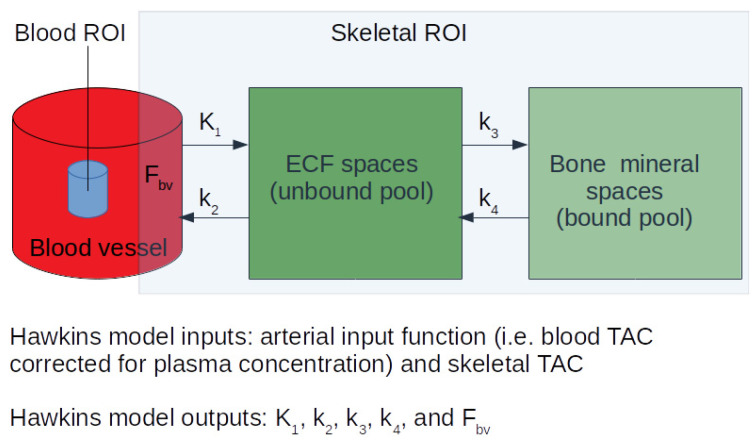
The two-tissue compartment Hawkins model was adapted from Hawkins et al. [33] and Azad et al. [34] to analyze bone studies using [^18^F]NaF PET. K_1_ (units: mL min^−1^ mL^−1^) describes the unidirectional clearance of fluoride from plasma to the whole of the bone tissue, k_2_ (units: 1/min) describes the reverse transport of fluoride from the ECF compartment to plasma, k_3_ and k_4_ (units: 1/min) describe the forward and backward transportation of fluoride from the bone ECF to the bone mineral compartment. In order to account for vascular [^18^F]NaF activity in the tissue region, a fifth parameter, fractional blood volume (F_BV_) is also sometimes included in the model as the time–activity curves of skeletal region of interest include small parts of blood vessels. PET = positron emission tomography; ECF = extracellular fluid.

Studies using the [^18^F]NaF PET technique are analyzed using a Hawkins’s two-tissue compartment model (Figure 1) [44] for the quantification of parameters of interest. A compartment is a volume or space within which the tracer rapidly becomes uniformly distributed and contains no significant concentration gradient. A compartment can have a physical interpretation, e.g., the intravascular blood pool, reactants and products in a chemical reaction, or substances that are separated by membranes. A less obvious physical interpretation may be a tracer that is metabolized or trapped by two different cell types in an organ, thus defining two populations of cells as separate compartments [18]. The Hawkins model describes the pharmacokinetics of [^18^F]NaF as it clears from the plasma into the unbound bone compartment (extracellular fluid (ECF)) and then the ionic exchange of the fluoride with hydroxyl groups in hydroxyapatite on the surface of the bone matrix to form fluorapatite preferentially at sites of osteoblastic and osteoclastic activity and newly mineralizing bone [45,46,47], as shown in Equation (1):

Ca_10_ (PO_4_)_6_(OH)_2_ + 2F^−^ => Ca_10_(PO_4_)_6_F_2_ + 2OH^−^ (1)

To obtain a dynamic PET-CT scan, participants are positioned supine with the scanner start time as the reference time. A computed tomography (CT) scan is performed first, followed by a PET scan. The tracer is administered at t = 10 s injected over 10 s. A 10 mL flush is injected at t = 20 s injected over 10 s. The images are acquired in list mode and re-binned at different frame durations during image reconstruction, along with corrections for scatter, random, deadtime and attenuation. Ordered subset expectation maximization or filtered back projection methods in 2D/3D mode can be used for image reconstruction, even though there may be some quantitative differences between the K_i_ results from images reconstructed using these two methods [48]. These images are then corrected for radioactive decay and used to obtain tissue time–activity curves (TAC) and/or and an image-derived arterial input function (IDAIF). The two inputs required for the Hawkins model are: (a) a TAC describing the tracer activity over time within the skeletal volume of interest (VOI) drawn on the dynamic PET scan images, and (b) an arterial input function (AIF) describing the tracer activity over time within the artery feeding the blood plasma to the skeletal VOI [18,44,49]. The AIF can be obtained in various ways, for example, by using continuous arterial blood sampling [50], venous sampling at early time points after warming the hands to 43 °C [51,52], or using venous blood sample obtained between 30–60 min post injection to calibrate image-derived time–activity curves of the blood [36]. Our present methods depend on arterial and venous concentrations becoming equal by 30 min after tracer injection and do not require heating the hands at 43 °C to obtain arterialized venous blood [33,36,53,54]. The blood AIF calibrated against the blood samples is then corrected for plasma-to-whole blood ratios measured from the blood data. These inputs can be fitted to the model using various different approaches [55], including nonlinear regression (NLR). The outputs of the model are individual rate constant parameters describing the transport of [^18^F]NaF between the blood plasma, extracellular fluid and bone mineral compartments (Figure 1). The physiological relevance of each of these exchange parameters is described in detail in the following sections.

## 2. K_i_

The K_i_ parameter represents the net plasma clearance of [^18^F]NaF tracer to the bone mineral space (Figure 1). It is calculated using Equation (2):

K_i_ = K_1_ × k_3_/(k_2_ + k_3_) mL min^−1^ mL^−1^ (2)

where, K_i_ is the product of K_1_ and the fraction of the tracer that undergoes specific binding to the bone mineral [k_3_/(k_2_ + k_3_)]. K_i_ is often referred to as the plasma clearance to the bone mineral compartment, or by some authors as the bone metabolic flux [40] or rate of bone turnover [34]. It can be understood as follows: a measured value of K_i_ of 0.01 mL min^−1^ mL^−1^ means that the quantity of [^18^F]NaF cleared to each milliliter of bone in one minute is equal to the amount of contained in 0.01 mL of plasma.

Bone histomorphometry studies following double tetracycline labeling [2,3] are an important method of validating the use of [^18^F]NaF PET in the investigation of metabolic bone diseases. Histomorphometric analysis provides both structural and remodeling bone parameters [30,31]. The remodeling parameters can be static or dynamic. Static remodeling parameters include osteoblast and osteoclast activities measured as a percentage of bone surface area, osteoid thickness, bone volume measured as a percentage of total tissue volume, osteoid volume measured as a percentage of bone volume, and eroded surface measured as a percentage of the bone surface. Dynamic remodeling parameters include bone formation rate and mineralizing surface, both measured as a percentage of bone surface area, activation frequency per year, and mineralization lag time [2,3].

In a bone histomorphometry study at the lumbar spine (L_1_–L_4_) in 26 renal dialysis patients, Aaltonen et al. [2] demonstrated strong correlations between K_i_ and most of the histomorphometric parameters, including activation frequency (r = 0.60; *p* = 0.002), mineralized surface per bone surface (r = 0.55; *p* = 0.003), and osteoblast per bone surface (r = 0.49; *p* < 0.01), osteoclast per bone surface (r = 0.62, *p* < 0.001), and bone formation rate per unit bone surface area (r = 0.63; *p* < 0.001). These results support an earlier study [16], which also found a significant correlation between the net uptake of fluoride ions by PET and bone formation at the iliac crest measured by histomorphometry. Therefore, in light of these results, K_i_ can be considered to be a measure of bone formation rate, or the bone remodeling that reflects metabolic activity, i.e., a combination of osteoblastic and osteoclastic activities. Another study by Piert et al. [56] correlated K_flux_ (K_flux_ = K_i_ × plasma concentration of stable nonradioactive fluoride) with bone mineral apposition rate (MAR). However, it is important to note that BFR seems a more appropriate variable to correlate with K_i_ because MAR (units: μm/day) is the maximum rate of daily increment of mineralized bone seen in a section of bone studied under the microscope, while BFR is the product of MAR and the area of active bone formation. BFR takes into account the percentage of the bone surface area undergoing active mineralization in the section under study, unlike the MAR, which might be large, but may be only a single small area of bone formation in the total area under study.

Even though dynamic PET studies of bone using [^18^F]NaF PET are complicated by the need to obtain arterial blood information, they provide invaluable information that is not possible to obtain with the conventional standardized uptake value (SUV) commonly used to quantify PET studies. Unlike SUV, the K_i_ value provides a local measurement in bone independent of any physiological changes in other parts of the body and hence represents the true change occurring at the measurement site unaffected by changes at other skeletal sites. In contrast, SUV measurements are influenced by the fact that a finite amount of administered tracer is shared across many skeletal sites in the whole body, and therefore are less specific to the measurement site than K_i_ [57,58]. For this reason, there are circumstances, such as the investigation of treatments that have a powerful effect on whole skeleton bone metabolism or the treatment of extensive metastatic bone disease or Paget’s disease, in which SUV and other types of bone uptake measurements are less reliable measures of biological change than K_i_.

In osteoporosis, anabolic treatments such as teriparatide tend to increase bone formation and therefore increase K_i_ [36] and antiresorptive treatments such as risedronate and other bisphosphonates tend to reduce bone resorption and therefore decrease K_i_ [33]. This is due to the changes in the activation frequency of newly forming bone [30,31]. A reduction in the potential number of sites for bone remodeling activity associated with a reduction in bone mass (particularly the reduced mass of trabecular bone) suggests there may be a benefit in applying a correction for bone surface area [59,60,61,62]. For example, this may explain why K_i_ values at the lumbar spine in postmenopausal osteoporotic women were lower than in nonosteoporotic controls despite the fact that osteoporosis is associated with a state of increased bone turnover [13]. Other studies have shown that bone turnover is an independent predictor of bone loss and fracture risk in osteoporosis [1,11,63,64] and that measurements of bone turnover can predict bone loss much earlier than the actual decrease in bone mineral density (BMD) [65]. High bone turnover increases fracture risk and bone loss by increasing the remodeling activity, leading to a deterioration in local bone microarchitecture, decrease in bone quality, structural weakness and increased susceptibility to fracture [4,6,7,8,66]. It is important to examine changes in bone turnover in response to treatment so its role in the quality of newly formed bone is fully appreciated. Studies show that the changes in bone turnover correlate well with changes in BMD and fracture risk [11,64,67,68,69,70,71,72,73,74,75,76] and it is the changes in bone turnover rather than the increase in BMD observed with antiresorptive therapies that explain much of the reduction in osteoporotic-related fracture risk [67,68,70].

Measurements of K_i_ may also have a role in the investigation of metastatic bone disease. In a recent study reported by Azad et al. [40], K_i_ was able to differentiate clinically progressive disease and nonprogressive disease more reliably than SUV measurements after 8 weeks of endocrine treatment in bone-predominant metastatic breast cancer patients. However, the authors suggested that larger patient groups under different therapy regimes are required to validate these findings further.

## 3. K_1_

The parameter K_1_ represents the [^18^F]NaF plasma clearance to the ECF space (Figure 1) via capillaries and is a measure of the local rate of bone perfusion. It is measured in terms of the volume of plasma cleared of tracer per unit time per volume of tissue, units: mL min^−1^ mL^−1^, and is related to regional blood flow by Equation (3):

K_1_ = E × Q × (1 − PVC) mL min^−1^ mL^−1^ (3)

where E is the unidirectional single-pass extraction efficiency of whole blood [^18^F]NaF, which is often assumed to approach 100% [77], Q is regional blood flow and PVC is the packed cell volume. [^18^F]NaF is suitable for this role because the fluoride ion has low atomic mass and is highly diffusible. At low to moderate flow rates (less than or equal to 0.16 mL min^−1^ mL^−1^), the bone perfusion measured using [^18^F]NaF is in good agreement with measurements made using [^15^O]H_2_O, the recognized gold standard for radionuclide blood flow studies [78,79]. However, at higher blood flow there is insufficient time for the [^18^F]NaF tracer to equilibrate with surrounding tissues and K_1_ underestimates true bone perfusion. K_1_ values measured at skeletal sites such as the lumbar spine (which is a highly metabolically active site) in healthy or osteoporotic subjects have been shown to have values lower than 0.16 mL min^−1^ mL^−1^ [13,34,50]. Therefore, K_1_ measurements of regional skeletal bone perfusion using [^18^F]NaF PET are usually considered a good approximation to regional bone blood flow.

Bone perfusion plays a crucial role in maintaining bone health [80,81,82,83,84,85,86,87], influencing both osteoblastic and osteoclastic activity [80,88] and bone remodeling [89], which is associated with an age-related increase in the rate of bone loss, osteoporosis and fracture risk [19,87,89,90]. Reeve et al. [89] reported a correlation between whole skeletal bone blood flow and MAR measured using bone biopsy. K_1_ also correlated with the skeletal influx rate of calcium in osteoporotic patients [89]. Even though the exact mechanism between bone metabolism and bone perfusion is not known, to a certain extent the impairment in vascular nitric oxide signaling and the vasodilator prostaglandin PGI2 [80,91,92] are considered responsible for the reduced bone perfusion observed with increased age. 

The reduction in bone perfusion is also associated with greater bone loss [87] and there is a direct relationship between bone perfusion and bone remodeling activity [56,89]. A reduction in bone blood flow with age has been observed in osteoporotic subjects when compared with healthy controls [83,93]. Another study in postmenopausal women with and without osteoporosis in the lumbar spine [13] showed no significant differences in K_1_, probably due to the large precision error in the measurement of K_1_ reducing the statistical power of the study to detect these differences. However, lower values of K_1_ were reported in the normal femoral head [94] and distal humerus [16,50] as compared to that in normal vertebra [50], which is likely to be one of the reasons for the lower bone metabolism at the hip and humerus compared to the lumbar spine. These results were supported by another study that showed lower values of K_1_ at the hip compared to the lumbar spine, suggesting that lower bone blood flow is an important factor explaining the differences in K_i_ at different skeletal sites [95]. Furthermore, the changes in marrow composition with ageing, specifically a decrease in functioning red bone marrow and its replacement by marrow fat, may be associated with reductions in bone perfusion. In adults, functioning red marrow is largely confined to the spine, and this may be a factor explaining the differences in K_1_ and therefore K_i_ between the hip and lumbar spine [95].

## 4. k_2_ and k_3_

The parameters k_2_ and k_3_ describe the backward and forward rate of [^18^F]NaF transfer from the bone extravascular compartment to plasma and bone mineral, respectively (Figure 1). Although the exact physiological meaning of these parameters is not clear, they reflect the overall extraction efficiency of the tracer from the extravascular compartment to bone mineral. In studies measuring response to treatment, antiresorptive treatments (such as risedronate) have been shown to increase k_2_ **[33]**, in turn affecting the concentration of tracer in the extravascular tissue space (Figure 1). In addition, k_2_ was found to be of particular interest in Pagetic bone, where the ECF compartment is considered more complicated than in normal bone [50]. Cook et al. [61] observed a lower value of k_2_ and a higher value of k_3_, with both factors contributing to the increased bone turnover. Pagetic bone often has its marrow space replaced by fibrous tissue, and it is possible that the [^18^F]NaF tracer that enters the extracellular fluid space may be less available for return to plasma.

## 5. k_4_

The parameter k_4_ describes the backward rate of [^18^F]NaF transfer from bone mineral to the extravascular compartment (Figure 1). The nonzero value of k_4_ reported by many studies reflects the fact that some of the tracer in the bone mineral space is only weakly bound and can diffuse back into the ECF space. The observed values of k_4_ are small (typically 0.01/min), corresponding to a tracer half-life in the bone mineral space of around 70 min [53,54]. Therefore, some methods of scan analysis assume the value of k_4_ is zero (for example, when using Patlak analysis [96] to calculate K_i_), an assumption that can lead to the reported values of K_i_ being lower by up to 25%. Puri et al. [95] reported no significant differences in the k_4_ values measured at lumbar spine and hip. In other studies, the impact of k_4_ was studied from a technical perspective to correct K_i_ measurements for the efflux of tracer from bone [41,53,54]. These will not be discussed further here.

## 6. K_1_/k_2_

The ratio K_1_/k_2_ can be understood as the effective volume of distribution of tracer in the ECF space (Figure 1). K_1_/k_2_ describes the fraction of the skeletal VOI used to create the bone tracer time activity curve occupied by the bone ECF compartment, assuming a passive diffusion of fluoride between plasma and ECF. This is not an unreasonable assumption for fluoride with such a small atomic weight. The ^18^F^−^ ion is also known to bind with hydrogen atoms, creating electrically neutral hydrogen fluoride (HF), which is a small and highly diffusible molecule that is able to cross the cell membrane. The [^18^F]NaF in the bone ECF compartment may be less available for exchange with other compartments due to binding with marrow spaces leading to limited access to the bone mineral surface [97]. A study by Cook et al. [50] reported values of K_1_/k_2_ corresponding to 48% of VOI volume in vertebral and 34% in humeral bone, which was in concordance with the earlier estimates in canine tibiae, which were 10% for bone ECF space alone and 24% for bone marrow ECF [98]. These findings support the fact that the ECF spaces are larger in trabecular-rich bone containing more marrow than in cortical bone.

Puri et al. [95] reported three-fold lower values of K_i_ at the hip compared to the lumbar spine and argued that one of the reasons (other than the differences in the K_1_ mainly due to the differences in marrow fat composition [84]) might be the significantly larger value of K_1_/k_2_ in vertebrae than in hip. This may be due to the presence of more functioning red marrow in the lumbar spine, where a greater volume fraction of the bone ROI may be accessible to fluoride ions than at the hip, where the bone marrow is largely fatty. This suggests the presence of a very small ECF space at the hip, which holds a relatively small amount of tracer available for uptake by bone mineral and might be an additional reason for lower K_i_ at the hip site. In another study, a decrease in the ratio K_1_/k_2_ at the lumbar spine in osteoporotic subjects treated with risedronate for 6 months suggested a smaller bone ECF space after treatment, although this did not reach statistical significance [33]. The authors argued that this finding might be because of an increase in BMD at the site, which could be mainly due to the filling of the remodeling space [99,100]. The K_1_/k_2_ parameter is also reported to be significantly greater in Pagetic bone and suggests larger bone and nonbone ECF spaces [61], which along with an increase in delivery and clearance to total bone tissue, could lead to an overall increase in clearance of [^18^F]NaF to the bone mineral compartment.

## 7. k_3_/(k_2_ + k_3_)

The ratio of k_3_ and k_2_ + k_3_ represents the fraction of tracer delivered by bone blood flow to the ECF space that binds to the bone mineral compartment and is equal to K_i_/K_1_ (Equation (2)). Puri et al. found no statistically significant differences between the values of k_3_/(k_2_ + k_3_) at the lumbar spine and hip in healthy postmenopausal women [95]. In studies measuring response to treatment, anabolic treatments (such as teriparatide) tend to increase k_3_/(k_2_ + k_3_) [36] and antiresorptive treatments (such as risedronate) tend to decrease k_3_/(k_2_ + k_3_) **[33]**, in turn affecting the fraction of tracer that underwent specific binding to bone mineral space (Figure 1). These results suggest that the ratio k_3_/(k_2_ + k_3_) may be an alternative measure of treatment response. Considering that the ratio of the treatment response divided by the precision error was highest for k_3_/(k_2_ + k_3_) among all the Hawkins’s model parameters [43], and that this ratio represents the fraction of tracer that undergoes binding to the bone mineral and thus of treatment effect [33,34,37], the measurement of changes in k_3_/(k_2_ + k_3_) may be considered one of the most effective means (alongside the K_i_ parameter) of using [^18^F]NaF dynamic PET scans to noninvasively investigate changes in bone formation rate.

## 8. Changes in K-Parameters in Response to Treatment

Several reports have described studies using [^18^F]NaF PET/CT to investigate the effect of osteoporosis treatments on bone turnover in postmenopausal women. Frost et al. examined the effect of 6 months treatment with the antiresorptive agent risedronate in 18 women (mean age 67 y) with low bone density at the spine or hip [33]. Mean vertebral K_i_ decreased by 18.4% (*p* = 0.04) with a similar decrease in the bone formation marker bone-specific alkaline phosphatase. The ratio k_3_/(k_2_ + k_3_) decreased by 18.1% (*p* = 0.02) and k_2_ increased by 58% (*p* < 0.05). No other kinetic parameters showed statistically significant changes. Subsequently, the same group examined the effect of 6 months treatment with the bone anabolic agent teriparatide (TPT) in 18 postmenopausal women with osteoporosis [36]. Mean vertebral K_i_ increased by 23.8% (*p* = 0.0003) with a similar increase in k_3_/(k_2_ + k_3_) (*p* = 0.0006). No other kinetic parameters showed statistically significant changes. Interestingly, the change in spine SUV was only 3% and was not statistically significant (*p* = 0.84). However, SUV increases of 36.9% in the femoral shaft (*p* = 0.0019) and total hip (*p* = 0.032) were significant. As mentioned previously, this finding demonstrates the importance of measuring K_i_ as well as SUV in patients with areas of highly elevated bone turnover in the skeleton, whether localized as in Paget’s disease [101] or extensive metastatic bone disease, or diffusely across the entire skeleton as in TPT treatment. In such patients, the arterial plasma concentration of tracer is depressed and SUV measurements can give a false impression of true regional change [58]. 

## 9. Precision Errors in Measuring K-Parameters

The precision error of each parameter in the Hawkins model expressed as the coefficient of variation (Table 1) and the treatment response expressed as the percentage change between baseline and 6 months after treatment with the bone anabolic agent teriparatide were presented by Blake et al. [43]. Parameters with a large response to treatment and a small precision error are the most sensitive parameters for measuring response to treatment and require the least number of subjects to reach a statistically significant difference in a drug trial. In the teriparatide study mentioned above, the only parameters with sufficient precision and treatment response to be clinically useful were K_i_ and k_3_/(k_2_ + k_3_). The limitation of the other parameters (K_1_, k_2_, k_3_, k_4_, and F_bv_) is that their precision errors are around 30% or greater [43], which is too poor to make them useful in most studies.

## 10. Conclusions

In conclusion, this review discusses kinetic indices obtained from dynamic [^18^F]NaF PET bone studies which have allowed us to understand local bone physiology at various skeletal sites at the cellular level before structural changes in bone may be detected, which may be useful for developing and testing bone-active drugs in the future.

## Figures and Tables

**Table 1:** Precision of [^18^F] sodium fluoride PET kinetic parameters [43] are listed in this table. %CoV results for SUV were 11% (9–14%). %CV: coefficient of variation; 95% CI: 95% confidence interval.

	k_1_	k_2_	k_3_	k_4_	F_bv_	k_3_/(k_2_ + k_3_)	k_i_
	mL min^−1^ mL^−1^	min^−1^	min^−1^	min^−1^			mL min^−1^ mL^−1^
%CV (95% CI)	36% (29–45%)	52% (42–67%)	28% (23–36%)	33% (27–42%)	55% (45–70%)	19% (15–24%)	15% (12–19%)

## Data Availability

Not applicable.
